# Pitfall trap design affects the capture efficiency of harvestmen (Opiliones) and millipedes (Diplopoda)

**DOI:** 10.1002/ece3.7820

**Published:** 2021-06-28

**Authors:** Slavomír Stašiov, Marek Čiliak, Michal Wiezik, Marek Svitok, Adela Wieziková, Andrea Diviaková

**Affiliations:** ^1^ Department of Biology and General Ecology Faculty of Ecology and Environmental Sciences Technical University in Zvolen Zvolen Slovakia; ^2^ Department of Applied Ecology Faculty of Ecology and Environmental Sciences Technical University in Zvolen Zvolen Slovakia; ^3^ Parlement européen Brussel Belgium; ^4^ Department of Ecosystem Biology Faculty of Science University of South Bohemia in České Budějovice České Budějovice Czechia

**Keywords:** body size, capture rate, fixing fluid, method selection, trap diameter

## Abstract

Pitfall trapping is one of the standard methods used for the capture of ground‐active arthropod groups. Despite being frequently used, the standardization of this method is problematic due to the large range of combinations of the individual parameters of pitfall traps with varying efficacy under different environmental conditions. We evaluated the effects of the trap diameter, the fixing fluid, and their combination on the capture efficacy for harvestmen (Opiliones) and millipedes (Diplopoda). We used pitfall traps with three different diameters: 3 cm, 5 cm, and 12 cm, filled with three types of fixing fluids (saturated fluid of NaCl, 10:1 mixture of 70% ethanol and glycerol and 4% formaldehyde). Altogether, 90 traps representing nine combinations of trap diameters and fixing fluid were placed on a mown meadow in spring and autumn intervals for a total of 45 days. We sampled 1,488 individuals representing 11 harvestmen species and 881 individuals representing 11 millipede species. Large (*d* = 12 cm) and medium (5 cm) traps captured significantly more millipede species and individuals than the small‐sized traps (3 cm). The same effect was observed for harvestmen species richness, whereas the medium traps (*d* = 5 cm) captured the highest mean activity of harvestmen. By analyzing the differences in the body sizes of the studied arthropods in relation to the trap diameter and fluid, we found that larger traps, as well as traps filled with NaCl solution, captured larger harvestmen more frequently than the other trap types. Our results revealed that the combination of larger traps (*d* = 5 and 12 cm) and formaldehyde was most effective in the capture of both studied groups. However, the disadvantage of formaldehyde is its toxicity.

## Graphical abstract

We evaluated the effects of the trap diameter, the fixing fluid, and their combination on the capture efficacy for harvestmen (Opiliones) and millipedes (Diplopoda). Our results revealed that the combination of larger traps (*d* = 5 and 12 cm) and formaldehyde was most effective in the capture of both studied groups. However, the disadvantage of formaldehyde is its toxicity.

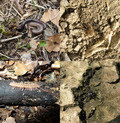

## 1 INTRODUCTION

Understanding changes in global biodiversity patterns requires large‐scale, long‐term monitoring. However, there might be significant constraints in the analysis of long‐term and spatially large data, especially when the methodology used differs (Gotelli & Colwell, 2001, 2011). The ability to make meaningful comparisons of the results of studies is severely hampered by great variation in the design of the sampling equipment and how it is used (Brown & Matthews, 2016; Dornelas et al., 2014; Fischer et al., 2010; Magurran et al., 2010). Data obtained by pitfall trapping in various studies also have limited comparability (Brown & Matthews, 2016).

Pitfall trapping was first used for the research of epigaeic fauna by Dahl (1896) and was further developed after the publication of Barber (1931) and Stammer (1948). It is now a well‐established and commonly used quantitative method of zoocoenosis surveys, including harvestman and millipede communities (Štrobl et al., 2019). Pitfall traps are simple to use, efficient, and inexpensive and allow for the continuous collection of specimens, including night foragers, thus overcoming interspecific differences in circadian activity rhythms (Koivula et al., 2003; Southwood, 1978; Törmälä, 1982; Ward et al., 2001).

This method is particularly advantageous for the capture of epigaeic macro‐ and megafauna with islet‐like dispersion and more pronounced differences in circadian activity (Stašiov, 2015). Pitfall traps can be used to generate an estimate of “activity density,” that is, the abundance of each species as a reflection of its activity during the sampling period and the density of the population in the sampled habitat. This method does not allow reliable estimation of species densities (Curtis, 1980; Lang, 2000).

The effectiveness of pitfall trapping for the capture of epigaeic fauna has been widely discussed (Luff, 1975; de Oliveira et al., 2019; Privet et al., 2020; Saska et al., 2013; Southwood, 1978; Topping & Sunderland, 1992). For example, the effectiveness of pitfall trapping was evaluated by capturing all individuals in a specific area by Adis (1979) and Petruška (1969). According to Adis (1979), the various factors possibly affecting the activity patterns of animals, and their numbers caught in pitfall traps, such as climatic conditions, vegetation cover, reproductive period, trap design, trapping fluid, and many others, differ in their influence on epigeal arthropod taxa. Petruška (1969) reported that carabids can escape from water and formalin traps when detergent is not used. Individuals of different sexes or ages may have unequal escape capabilities, which will be a source of inconsistent results. Banerjee (1970) and Müller (1984) used marking of individuals trapped in pitfall traps and their retrapping to calculate density using data of the relative abundance. According to Banerjee (1970), trapping of three species of diplopods with pitfall traps in areas with known densities follows a definite mathematical relationship. The number of each species trapped is density‐dependent up to a certain level: beyond that, the increased density levels are not proportionately reflected in the catches. Müller (1984) found that in carabids, pitfall trapping overestimates the number of captured males relative to females because they have higher epigaeic activity.

Pitfall trapping is a passive method. This method of collecting biological material has several advantages. In particular, the results obtained are not affected by the collector; it is a simple and effective method and not very labor‐intensive (Franke et al., 1988; Prasifka et al., 2007; Skvarla et al., 2014; Zou et al., 2012). Traps operate continuously from installation to collection. Therefore, it is possible to work simultaneously on different remote sites, and the results can be compared with each other.

Harvestmen (Opiliones) and millipedes (Diplopoda) are epigaeic organisms for which the trappability of variously modified pitfall traps has not been sufficiently investigated (Gerlach et al., 2009; Tourinho & Lo‐Man‐Hung, 2021). Therefore, the lack of information has motivated us to compare the effectiveness of some previously untried pitfall trapping modifications in capturing these two groups of epigaeic invertebrates.

The primary objective of our study was to test the hypothesis that when capturing harvestmen and millipedes by pitfall trapping, the compositional structure and abundance of their communities vary between traps with two different basic parameters (diameter size and type of fixing fluids). The research was conducted under mesophilic meadow conditions. We expected to find a higher species richness and abundance of both groups in larger traps. Traps with a larger diameter also have a larger circumference compared with smaller traps and thus a larger capture zone. For this reason, it can be assumed that they record more individuals and species than smaller traps. We also expected that larger species would be captured less in smaller traps than in larger traps because larger species can cross over smaller traps and not fall into them. These assumptions have already been confirmed in several groups of epigaeic invertebrates but not yet in harvestmen or millipedes. For example, Luff (1975) noticed that larger pitfall traps will always collect more individuals because the rate of encounter is closely related to the circumference of a trap. Work et al. (2002) found that larger pitfall traps with larger circumferences collected more individuals and more species of three studied taxa (Coleoptera: Carabidae and Staphylinidae; and Araneae: Lycosidae) and that smaller traps collected more small‐bodied carabid and staphylinid species and large traps collected more wolf spiders (Lycosidae) than smaller traps. According to Thiele (1977), higher catch rates of larger species in larger traps may reflect the positive correlation between body size and motility. We assumed that pitfall traps with different tested fixing fluids would also provide different data on the species richness and abundance of both groups due to their different effects (attractive or repellent) on these groups and probably also on individual species. The reason for the different capture efficiencies of the tested fixing fluids may also be their specific effect on the escape of epigaeic invertebrates from traps, which may be conditioned by their different densities or killing effects. McCravy and Willand (2007) revealed different effects (attraction/repellency) of several tested fixing fluids on the numbers and species of carabids captured by pitfall trapping. Pitfall traps containing the four preservatives that appeared to produce the strongest volatiles (acetic acid, ethanol, ethylene glycol, and propylene glycol) collected much greater numbers of carabids than those containing distilled water or brine. However, these authors also pointed out that these four preservatives may kill beetles more quickly, reducing the probability of escape. Schmidt et al. (2006) found that brine and ethanol–glycerin showed low capture efficiencies of arthropods compared with pure water and ethanol–water, presumably because their high specific density allowed them to float and thereby facilitated their escape.

## 2 MATERIAL AND METHODS

### 2.1 Study area

The study was conducted in the Štiavnické vrchy Mountains, which is a geomorphologic area of the West Carpathians in the central part of Slovakia. The bedrock consists mainly of andesites and rhyolites with scattered occurrences of conglomerates and shales. Soils are predominantly cambisols. A substantial part of the area has a moderately warm climate with a mean annual precipitation of 650–800 mm and a mean air temperature of 7–8°C (Miklós, 2003).

The research was conducted on a mown meadow at an altitude of 389 m situated on a distinctive steep slope with a western exposure, with a slope of 17–25°. The sampling site was located in the exclave of Kozelník village (48°30.807′N and 19°00.210′E).

The locality represents communities of mesophilic, lowland, and submontane meadows of the alliance *Arrhenatherion elatioris*. The ecological spectrum of their occurrence is relatively wide, which is closely related to their variability in species composition (Stanová & Valachovič, 2002), dominated by high‐grass species such as *Arrhenatherum elatius*, *Dactylis glomerata*, and *Festuca pratensis*, associated with relatively diverse groups of herbs including *Ranunculus acris*, *Trifolium pratense*, *Achillea millefolium,* and *Lychnis flos‐cuculi*.

### 2.2 Study design

Pitfall traps used to sample ground‐dwelling invertebrates were exposed in two intervals: 19.4–16.5.2009 and 16.9–2.10.2009. We used three different diameters of traps (3 cm with a depth of 5 cm, 5 cm with a depth of 7 cm, and 12 cm with a depth of 13 cm) and three different types of fixing fluids (saturated solution of NaCl (brine), 10:1 mixture of 70% ethanol and glycerin and 4% formaldehyde). The brine was used as a control, as it is not known to have any attractive or repellent attributes. In this manner, we established 9 different treatment combinations, each represented by 10 replicates (90 traps in total). All traps were installed in a rectangular grid made of 10 rows of 9 traps, with 3 m intervals between the traps and rows. The placement of particular traps and treatment combinations followed a random pattern.

Harvestmen were identified following Martens (1978) and Wijnhoven (2009), and millipedes were identified following Kocourek et al. (2017). Voucher specimens are stored with the first author who identified them.

### 2.3 Statistical analysis

Since epigeon sampling by pitfall traps usually covers several seasons due to high seasonal dynamics (e.g., Inyang & Emosairue, 2003; Pekár, 2002; Siewers et al., 2014), trap samples from spring and autumn sampling periods were pooled for each trap, and summary data on species richness and epigaeic activity were used in the analyses.

The effects of fixing‐fluid type and trap‐diameter size on species richness, epigaeic activity (the number of individuals captured), Shannon diversity index, and evenness of Diplopoda and Opiliones were evaluated separately for each taxonomic group using generalized linear models (GLMs). The GLMs involved the main effects of fluid type and trap‐diameter size and their interaction. Since the number of species and individuals are count data, the GLMs were fitted with a Poisson or quasi‐Poisson error distribution (to account for overdispersion) using a log‐link function. In the case of the Shannon index and evenness, we used a Gaussian error distribution with an identity‐link function to fit the GLMs. When global tests of the main effects or interactions yielded significant results (*p* < .05), pairwise comparisons among groups were performed using Tukey's test.

The GLMs were also used to assess differences in body size of captured Diplopoda and Opiliones among traps of differing diameters and fixing‐fluid type. Millipede total body length (Ilić et al., 2019) and harvestmen leg span (Lindtner et al., 2020) were used as characteristics of body sizes. Data on these traits were taken from Martens (1978) and Kocourek et al. (2017). The mean body sizes of millipedes and harvestmen weighted by the species abundances were calculated for each trap. GLMs with a gamma error distribution and inverse‐link function were fitted to the weighted means of body sizes. Again, the interacting effects of fluid type and trap‐diameter size were used as explanatory variables.

Multivariate analogue of analysis of variance with permutations—PERMANOVA (Anderson, 2001)—was applied to test for the effects of fixing‐fluid type and trap‐diameter size on the composition of millipede and harvestman assemblages. The Bray–Curtis index was used as a measure of dissimilarity, and the calculation of p‐values was based on 9,999 permutations (McArdle & Anderson, 2001). Species data were log‐transformed prior to the analysis to reduce the effect of dominant species. The effects of fluid type and trap‐diameter size on the composition of millipede and harvestman assemblages were visualized by using nonmetric multidimensional scaling (NMDS; Kruskal, 1964). The same procedure (Bray–Curtis index and log‐transformed species matrix) was applied to both species datasets.

Since it was expected that the response of the species captured in traps might be specific compared with the response of the taxonomic group as a whole, we performed indicator species analysis (Dufrene & Legendre, 1997) to determine whether some species were captured more frequently in specific types of pitfall traps characterized by the fluid type and diameter. The indicator value was calculated for each species as the product of the species relative frequency and relative average abundance within the particular treatment combination of traps. The significance of the indicative weight of species was tested by the Monte Carlo permutation test (9,999 permutations).

Analyses were performed in R (version 3.5.2; R Core Team, 2018) using the “multcomp” (Hothorn et al., 2008) and “labdsv” (Roberts, 2016) packages and the DISTLM v.5 program (Anderson, 2004).

## 3 RESULTS

During the two sampling periods, we collected 1,488 individuals of 11 harvestmen species and 881 individuals of 11 millipedes species (Table A1).

**FIGURE 1:**
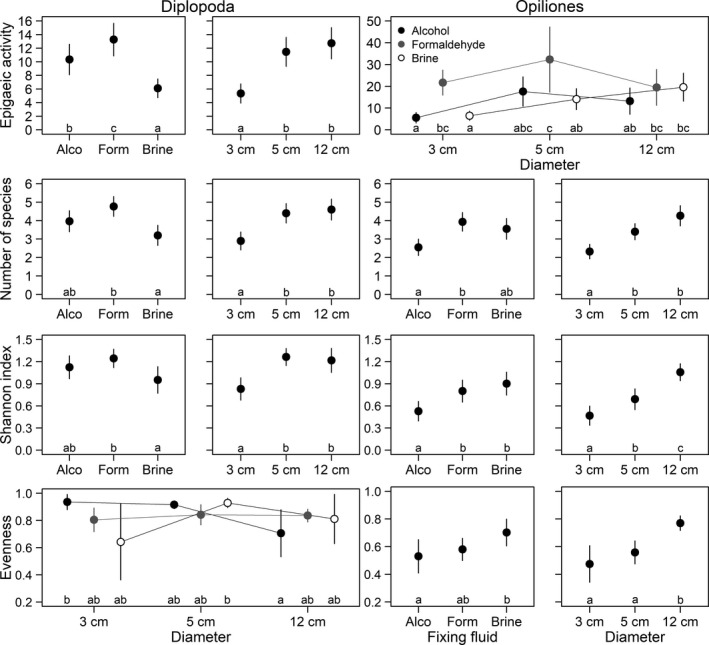
Comparisons of mean epigaeic activity, species richness, Shannon index of diversity, and evenness (points) of millipedes (left) and harvestmen (right) in relation to fixing‐fluid type used in traps, diameter of traps, or their interaction. Error bars represent 95% confidence intervals. Different letters indicate significant differences in means (*p* < .05) based on Tukey tests. In the case of a nonsignificant interaction between fixing‐fluid type and diameter size, the main effects were plotted separately

The effect of trap characteristics on species richness was very similar for both arthropod groups (Figure 1; Table 1). In both groups, the traps filled with formaldehyde captured the highest mean species richness. Among the three different diameter sizes of traps, the large‐ and medium‐sized traps captured significantly more species than the small‐sized traps. The same effects were observed for millipede epigaeic activity and the Shannon diversity index of millipede and harvestman assemblages; nevertheless, the highest mean harvestmen Shannon index was observed for the traps filled with brine. We found significant interacting effects of fixing‐fluid type and diameter size on harvestmen epigaeic activity and millipede evenness. In the case of harvestmen, we found significant independent effects of diameter size and fixing fluid on their evenness (Figure 1; Table 1).

**TABLE 1:** Overall differences in species richness, epigaeic activity, Shannon index, evenness, body length, leg span, and composition of millipede and harvestmen assemblages captured by pitfall traps in relation to diameter size and fixing fluid used (GLM with (quasi‐)Poisson, Gaussian, or Gamma error distribution and PERMANOVA with 9,999 permutations and Bray–Curtis dissimilarity)

	Diameter			Fixing fluid			Diameter* Fluid		
	*χ^2^* (*F*)	*D^2^* (%)	*p*	*χ^2^* (*F*)	*D^2^* (%)	*p*	*χ^2^* (*F*)	*D^2^* (%)	*p*
Species richness									
Diplopoda	15.71	21.93	<0.001	9.42	13.14	0.009	3.89	5.43	0.42
Opiliones	21.15	28.97	<0.001	10.5	14.38	<0.001	1.42	1.95	0.841
Epigaeic activity									
Diplopoda	28.69	29.37	<0.001	20.99	21.49	<0.001	2.1	4.29	0.089
Opiliones	8.01	12.33	<0.001	12.35	19	<0.001	3.24	9.98	0.016
Shannon index									
Diplopoda	11.47	19.15	<0.001	4.45	7.44	0.015	1.98	6.61	0.106
Opiliones	25.0	32.12	<0.001	10.6	13.74	<0.001	1.1	6.8	0.368
Evenness									
Diplopoda	3.19	6.33	0.047	1.25	2.48	0.293	2.96	11.76	0.025
Opiliones	10.4	19.35	<0.001	3.45	6.42	0.037	0.18	0.66	0.95
Body length									
Diplopoda	1.7	3.66	0.189	0.49	1.05	0.615	0.42	1.82	0.792
Leg span									
Opiliones	25.69	34.29	<0.001	7.84	10.47	<0.001	1.21	3.22	0.315

	pseudo‐*F*	*R* ^2^ (%)	*p*	pseudo‐*F*	*R^2^* (%)	*p*	pseudo‐*F*	*R^2^* (%)	*p*
Species composition									
Diplopoda	3.57	6.4	<0.001	8.76	15.6	<0.001	1.94	6.9	0.008
Opiliones	7.41	13	<0.001	6.11	10.7	<0.001	1.83	6.4	0.021

Note

Test statistics (*χ* ^2^, *F*, and pseudo‐*F*), deviance (*D* ^2^), variation explained (*R* ^2^), and corresponding probabilities (*p*) are given for each term and interaction.

**FIGURE 2:**
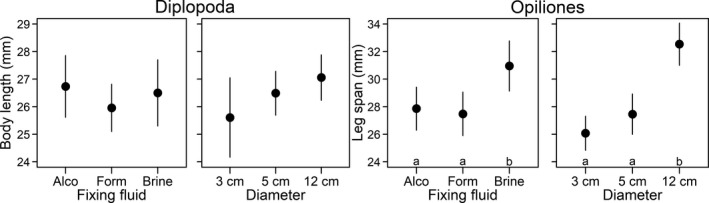
Comparison of the mean millipede body length and leg span of harvestmen in relation to fixing‐fluid type used in traps, diameter of traps, or their interaction. Homogeneous groups with statistically similar means resulting from Tukey's pairwise test are indicated by the same letter. Means are represented by black dots, and 95% confidence intervals are represented by vertical lines. Since the interactions between fixing‐fluid type and diameter size were nonsignificant, the main effects were plotted separately. In the case of Diplopoda, no significant differences in mean body length were found

We did not find any significant relationship between the trap characteristics and the mean body length of captured millipedes (Figure 2; Table 1). In contrast, both diameter size and fixing fluid showed significant independent effects on the mean leg span of captured harvestmen. The larger traps and traps filled with brine captured larger harvestmen species more frequently than the other trap types.

**FIGURE 3:**
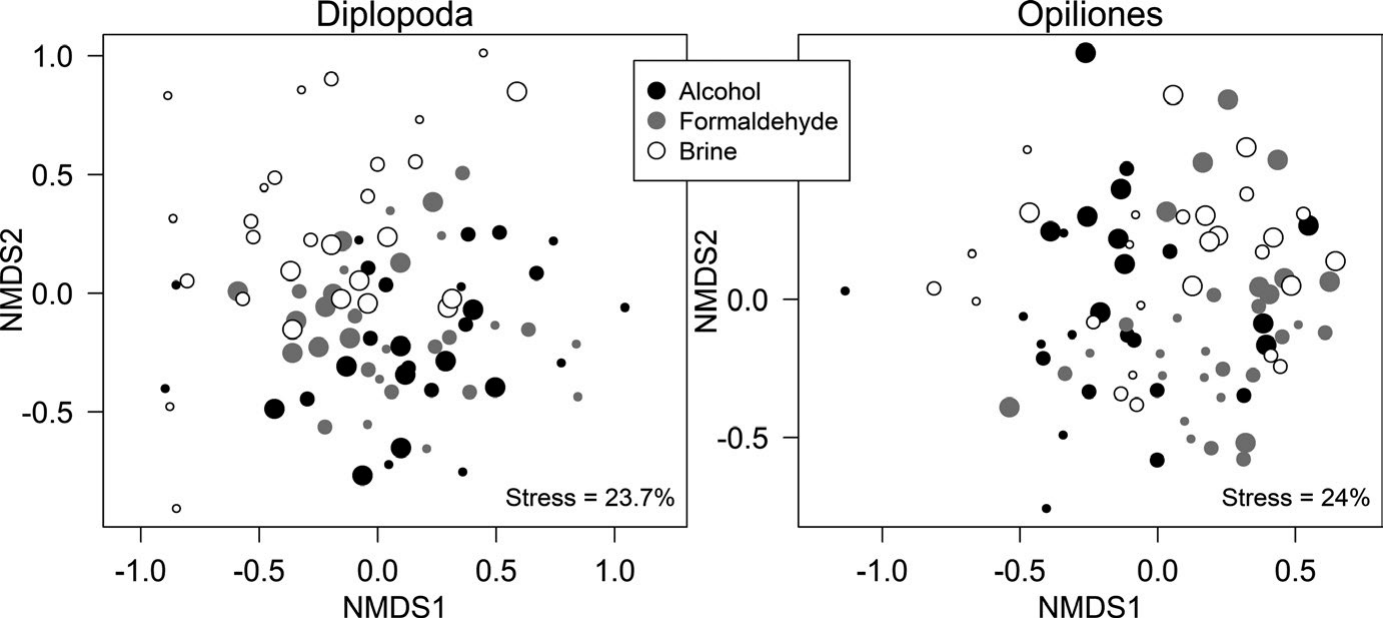
Nonmetric MDS ordinations of millipede (left) and harvestmen (right) assemblages captured by pitfall traps in relation to fixing‐fluid type (depicted by the different colors of the sample centroids—circles) and trap‐diameter size (depicted by the different sizes of the sample centroids, larger symbol = larger trap diameter)

We found significant interacting effects of fixing‐fluid type and diameter size on the community composition of both invertebrate groups (Table 1). Although diameter size explained the higher variation in harvestmen species composition compared with fixing‐fluid type, the fluid type had a greater influence on the millipede species composition in contrast to diameter size. These findings were visually supported by NMDS ordinations (Figure 3). In particular, millipede assemblages captured in the traps filled with brine were distinctly separated from those captured in the traps filled with alcohol (Figure 3 left). The differentiation of harvestmen assemblages was apparently caused by the trap‐diameter size, with the larger traps (regardless of the fluid type) being clearly separated from the small ones (Figure 3 right).

Indicator species analysis revealed six millipedes and five harvestmen species significantly associated with one of the fixing‐fluid types in combination with the diameter size of the traps (Table 2). Most of them were significantly dominated in the medium and large traps filled with formaldehyde. None of the harvestmen species was significantly associated with the combination of small and ethanol‐filled traps. Two species, *Z. crista* and *L. palpinalis*, were more common in brine‐filled traps, whereas *L. proximus* was the only species associated with ethanol‐filled traps. In millipedes, five species were associated with formaldehyde fluid across a range of trap diameters, while higher catch rates were associated with alcohol fluid only in *L. proximus*.

**TABLE 2:** List of indicator species for 9 combinations of pitfall traps differing in diameter size (3 cm, 5 cm, and 12 cm) and fixing‐fluid type (alcohol, formaldehyde, and brine)

Species	IndVal (%)	3 cm Alc	5 cm Alc	12 cm Alc	3 cm For	5 cm For	12 cm For	3 cm Bri	5 cm Bri	12 cm Bri
Diplopoda										
*Megaphyllum unilineatum*	30.5***	1/1	10/4	1/1	4/3	6/4	**32/10**	9/5	27/9	15/7
*Mastigona bosniensis*	23.6*	0/0	1/1	4/4	1/1	6/4	**11/6**	0/0	0/0	5/3
*Leptoiulus proximus*	22.9*	3/3	**31/10**	25/7	7/5	16/8	17/7	5/5	17/8	14/8
*Brachydesmus superus*	22.5*	0/0	0/0	0/0	**3/3**	1/1	0/0	0/0	0/0	0/0
*Polydesmus denticulatus*	21.8*	0/0	8/3	0/0	7/4	**18/4**	0/0	0/0	0/0	0/0
*Unciger transsilvanicus*	20.1*	4/3	5/4	6/4	7/5	10/5	**15/9**	4/3	10/5	6/4
*Polydesmus complanatus*	19.5	11/6	38/10	68/8	54/10	66/10	66/10	2/2	5/5	28/9
*Megaphyllum projectum*	14.7	6/4	10/5	9/5	1/1	6/4	2/2	0/0	0/0	0/0
*Ommatoiulus sabulosus*	14.5	1/1	6/3	3/3	2/2	3/2	6/4	0/0	1/1	7/6
*Glomeris hexasticha*	12.7	10/7	16/6	16/8	6/6	13/8	12/5	7/4	11/6	10/6
*Strongylosoma stigmatosum*	11.4	0/0	3/2	4/2	0/0	0/0	0/0	0/0	0/0	0/0
Opiliones										
*Zachaeus crista*	38.5***	1/1	6/3	9/6	1/1	7/4	20/8	3/3	9/4	**35/10**
*Oligolophus tridens*	30.3**	0/0	0/0	10/4	6/4	5/5	**28/8**	1/1	13/5	11/3
*Nemastoma lugubre*	27.6**	4/3	2/2	3/2	16/7	**29/8**	11/6	1/1	6/5	12/6
*Astrobunus laevipes*	24.9**	43/9	154/10	94/10	182/10	**266/10**	109/10	42/9	85/10	91/10
*Lacinius ephippiatus*	20.3	0/0	2/2	8/3	2/1	1/1	8/6	7/3	21/6	25/6
*Lophopilio palpinalis*	19.1*	1/1	6/4	6/5	8/5	9/6	10/5	4/3	4/4	**18/7**
*Trogulus nepaeformis*	12.9	0/0	3/3	0/0	0/0	2/2	1/1	0/0	1/1	0/0
*Trogulus tricarinatus*	10.9	1/1	2/2	1/1	2/2	4/2	6/4	0/0	2/2	4/4
*Platybunus bucephalus*	10.0	0/0	1/1	0/0	0/0	0/0	0/0	0/0	0/0	0/0
*Rilaena triangularis*	10.0	0/0	0/0	0/0	0/0	0/0	1/1	0/0	0/0	0/0
*Dicranolasma scabrum*	5.00	0/0	0/0	1/1	0/0	0/0	1/1	0/0	0/0	0/0

Note

Indicative values (IndVal) and epigaeic activity/number of traps within the combination are given for each species. The combination for which the species is indicative is in bold. Species are listed according to the descending indicative value within the particular taxonomic group. Asterisks represent statistically significant results of the Monte Carlo permutation test (9,999 permutations).

* *p* < .05

** *p* < .01

*** *p* < .001.

## 4 DISCUSSION

### 4.1 Influence of fixing fluid

Apart from the placement design, the fixing fluid used in traps can also strongly influence catches of invertebrates (Holopainen, 1992; Knapp & Růžička, 2012; Koivula et al., 2003; Lövei & Sunderland, 1996; Luff, 1975). The choice of a killing preservative has been a source of considerable debate in the literature because depending on the aims of the research, the ability to standardize the killing preservative is probably less easily achieved than for the other design features. First, there are different expectations based on whether the objective is to determine the assemblage (composition and abundance) or only to list the species present (presence/absence). Those wishing to collect material for genetic purposes will have different priorities than those solely wishing to investigate morphological features. The choice of killing preservatives can also be influenced by efforts to protect the environment, as some killing preservatives are toxic (Brown & Matthews, 2016). For example, formaldehyde is highly toxic. Different fluids may attract or repel particular species, which may bias the catch rates and sampling results (Knapp et al., 2016).

The results of this research revealed that among the tested fixing fluids, formaldehyde proved to be the most effective in capturing the highest number of species and individuals. The reason for this could be the higher attractiveness of this fluid for species from the studied groups. Some authors believe that the most commonly used fixing fluid (10% formaldehyde) is attractive to some invertebrates due to its vapors, but to others, it is repulsive (Greenslade, 1964). However, this assumption has not yet been confirmed in practice. Pinto‐da‐Rocha et al. (2007) also studied the impact of trapping fluids on the behavior of harvestmen and the effectiveness of the traps. According to them, bait traps (e.g., with invertebrate carrion) are much more effective than formalin traps for some harvestmen species. However, even formalin traps can work similarly to bait traps because they also catch mammals, amphibians, and slugs, which rot quickly with a bad smell, affecting catches of target arthropods (Zou et al., 2012). The higher efficiency of formaldehyde compared with the other tested fixing fluids could also result from the lowered escape rate of fallen invertebrates from formalin traps due to their relatively rapid death by this fixing fluid.

The disadvantage of formaldehyde is that it is a toxic fluid and produces toxic waste as well as potentially contaminating habitats. The same is true for ethylene glycol, which can block alcohol receptors in vertebrates and intoxicate them. Therefore, these fixing fluids cannot be recommended for pitfall trapping and need to be replaced by harmless alternatives.

### 4.2 Influence of the diameter size of the trap

Our results revealed that both studied groups were best sampled by using the traps with the largest and medium diameters. The diameter of the trap opening is a basic characteristic of the pitfall trap size because it influences the rate of capture of specimens. According to Luff (1975), the capture results were affected by the trap size (with small traps being more efficient in catching small beetles, while large traps were more efficient in catching larger beetles). Koivula et al. (2003) showed that larger traps (9 cm mouth diameter) are more efficient in collecting ground beetles than small traps (6.5 cm) in meadows in Finland. Large traps were also shown to be optimal in collecting spiders in a western Australian Jarrah forest (Brennan et al., 1999). Abensperg‐Traun and Steven (1995) found that only pitfall traps with large diameters (8.6 and 13.5 cm) caught all large‐bodied ant species.

Between the two compared attributes of pitfall traps, diameter size had a greater impact on harvestmen compared with millipedes, as it explained more variation in harvestmen species composition compared with fluid type. This may have been due to a larger range of harvestmen body sizes (range of the 2nd pair of walking limbs) ranging from 18.2 mm (smallest species) to 80.3 mm (largest species) compared with the size of millipedes (body length) ranging from 9 mm to 62 mm. Due to a larger range of body sizes (62.2 compared with 53 mm), the diameter size had a greater impact on trapping efficiency for harvestmen than for millipedes. Moreover, 9 out of 11 recorded millipedes had a body length of up to 30 mm, which was also the diameter of the smallest tested trap. In contrast, only 4 of 11 harvestman species had a body size of up to 30 mm, indicating that most species probably found it easier to avoid the smallest traps compared with the millipedes. Another reason may be the different shapes of harvestmen and millipede bodies, which allows the body of a harvestman (including the legs) to occupy a significantly larger area than the body of the same‐sized (long) millipede. Therefore, harvestmen are more likely to cross over a smaller trap and not fall into it than the same‐sized millipedes. The cause of the greater impact of the diameter size of traps on trapping efficiency for harvestmen compared with millipedes could also be their significantly different mode of movement (speed, step length, etc.). Harvestmen are more mobile than millipedes, and their visual organs are located higher above the soil surface. Due to these abilities, they are probably more likely to avoid smaller traps than millipedes.

Our results revealed that larger traps, with a diameter size of 12 cm, proved to be effective in catching both groups. Altogether, six species (three for each group) were indicative of large traps. Two harvestmen and two millipedes were indicative of medium traps (5 cm diameter). *Nemastoma lugubre*, found predominantly in medium traps, was the second smallest recorded species (maximum 14 mm), and *A. laevipes* was the third smallest recorded species of harvestmen (25 mm); similarly, millipedes *L. proximus* and *P. denticulatus* reach body sizes not exceeding 30 mm. There was only one millipede species indicative of small traps, and again, it was the smallest recorded species with a maximum length of 9.5 mm. Based on our results, the small traps proved to be less effective and biased the catch toward species with small body sizes. In particular, long‐legged harvestmen are likely to run over and evade a smaller diameter pitfall trap. Skvarla et al. (2014) and Collett and Fisher (2017) also recorded a lower efficiency of small pitfall traps in catching larger invertebrates.

The smallest of the tested pitfall traps were not efficient in capturing millipedes. The body length of most recorded millipede species (7) exceeds 20 mm; hence, they may be capable of evading and escaping the small trap, as shown by Gerlach et al. (2009) in laboratory experiment with empty traps. Behavior patterns depend on visual and tactile inputs of the trap, the illumination, and the characteristics of the specimens themselves. Interestingly, the smallest millipede species were under‐represented in medium and large traps, suggesting a possible repelling effect of the larger trap containers on minute millipedes.

Even though confirmed to be the most effective within the tested trap‐diameter sizes, the use of large pitfall traps may have some disadvantages related to a collateral catch of invertebrates and vertebrates (some of which may be legally protected), whose bodies may attract other organisms to the traps and distort the data obtained by pitfall trapping (Brennan et al., 1999).

### 4.3 Evaluation of the efficiency of various types of pitfall traps

Analysis of both factors (diameter size of traps and fixing fluid) revealed that the combination of pitfall traps with a 12‐cm diameter size and formaldehyde fluid was the most effective at catching millipedes. The situation was different for harvestmen, where only one species was indicative of this combination, while surprisingly, two species were represented predominantly in medium traps filled with formaldehyde. This combination even recorded significantly more specimens of harvestmen compared with the combination of large traps and formaldehyde.

The effectiveness of different types of pitfall traps in harvestmen capture has also been tested in various habitats (spruce forest and field hedgerows) by Sechterová‐Špičáková (1989). She compared 8 types of traps with different shapes, materials, and diameters (from 18 to 38 cm). The author did not detect an effect of the type of pitfall trap on the quantity or quantity of catches. However, she found that a network‐based distribution of traps is more appropriate than a linear distribution to obtain objective quantitative data on harvestmen.

Brown and Matthews (2016) proposed that a “standardized pitfall trap for biodiversity monitoring” for the generation of long‐term and spatially large ecological datasets should implement the following design features: diameter—9–10 cm, fixing fluid—100 ml of a transparent, nontoxic fixing fluid such as propylene glycol. The capture results are, in addition to the diameter size of the pitfall traps and fixing fluid, also affected by the trap shape (Spence & Niemelä, 1994), material of the trap (Luff, 1975), fluid concentration, and detergent (Pekár, 2002), as well as by the cover used (Spence & Niemelä, 1994).

Several authors have compared pitfall trapping with other methods used for the capture of ground‐active arthropod groups. For example, comparisons among pitfall traps and litter bags placed above‐ or belowground indicated that aboveground litter bags most frequently succeeded in collecting certain groups of arthropods associated with moisture and sheltered areas, including centipedes (Chilopoda) and beetle larvae (Carabidae and Staphylinidae). Conversely, pitfall traps most often captured taxa considered active at ground level, such as adult carabids or harvestmen (Opiliones) (Prasifka et al., 2007). Blower (1970) studied the density and surface activity of millipedes in sycamore ash wood. Seven species were extracted by Tullgren funnels from samples of soil and litter over 5 years and were also caught in pitfall traps during an additional 2 years; four other species occurred occasionally in the traps. Branquart et al. (1995) researched woodlouse (Isopoda) and millipede assemblages in nine deciduous oak‐forest stands. The numerical size of the populations was estimated using two sampling methods, that is, extraction from soil samples (abundances) and pitfall trapping (activities). Although a good relationship was registered between abundances and activities estimated in spring and autumn, pitfall efficiency greatly depended on the taxa concerned. According to Curtis (1980), the data distortions and problems of interpretation explicitly described for pitfall trapping will apply to any sampling method.

The results of these and other studies suggest that a more objective knowledge of species composition and dominance of species in harvestman and millipede communities can be provided by a combination of several sampling methods due to their different trappability of individual species. In addition, the use of different sampling methods (e.g., sweep net and visual search) will also be important for inventory studies as well as for community ecology studies, as different methods sample different taxa (Churchill, 1993; Majer, 1997). Additionally, according to Knapp et al. (2020), not only different higher taxa but also different species from the same taxon can be collected efficiently with different sampling techniques; thus, the application of multiple (complementary) sampling techniques is recommended even for the sampling of a single higher taxon.

## 5 CONCLUSIONS

Our results revealed that both studied groups were best sampled by using traps with the largest (12 cm) and medium diameters (5 cm). They were shown to be similarly efficient (in terms of species richness and epigaeic activity), without significant overall differences in capture rates. Only the smallest traps were shown to be significantly less efficient and hence not suitable to representatively sample these two groups. The use of smaller traps for sampling arthropods may be preferable, especially in conditions where placing larger traps is problematic due to environmental constraints (such as shallow or rocky soils) or is of concern due to the possibility of catching protected species or collateral groups. It might also represent a cost‐effective method due to overall lower prices of the smaller containers and lower volumes of fixing fluids needed for a trap to operate. Although formaldehyde was shown to be the most effective at capturing both studied groups, it is a toxic fluid; therefore, it is necessary to replace it in pitfall traps with other harmless fixing fluids.

## CONFLICT OF INTEREST

None declare.

## AUTHOR CONTRIBUTIONS

**Slavomír Stašiov:** Conceptualization (lead); data curation (lead); investigation (lead); methodology (equal); supervision (lead); writing–original draft (lead); writing–review and editing (equal). **Marek Čiliak:** Formal analysis (lead); visualization (lead); writing–original draft (equal); writing–review and editing (equal). **Michal Wiezik:** Conceptualization (equal); investigation (equal); methodology (equal); writing–original draft (equal); writing–review and editing (equal). **Marek Svitok:** Formal analysis (equal); methodology (lead); writing–original draft (supporting); writing–review and editing (equal). **Adela Wieziková:** Investigation (equal); writing–original draft (supporting); writing–review and editing (supporting). **Andrea Diviaková:** Investigation (equal); writing–original draft (supporting); writing–review and editing (supporting).

## DATA AVAILABILITY STATEMENT

The data that support the findings of this study are openly available in figshare at https://doi.org/10.6084/m9.figshare.13985507.

## APPENDIX A

**TABLE A1:** List of recorded species and their spring/autumn epigaeic activities captured in pitfall traps differing in fixing‐fluid type and diameter size

Species	Fixing fluid			Diameter		
	Alcohol	Formaldehyde	Brine	3 cm	5 cm	12 cm
Diplopoda						
*Glomeris hexasticha* Brandt, 1833	39/3	28/3	26/2	23/0	37/3	33/5
*Mastigona bosniensis* (Verhoeff, 1897)	0/5	0/18	0/5	0/1	0/7	0/20
*Brachydesmus superus* Latzel, 1884	0/0	3/1	0/0	2/1	1/0	0/0
*Polydesmus complanatus* (Linnaeus, 1761)	46/71	103/83	0/35	33/34	42/67	74/88
*Polydesmus denticulatus* C. L. Koch, 1847	8/0	25/0	0/0	7/0	26/0	0/0
*Strongylosoma stigmatosum* (Eichwald, 1830)	7/0	0/0	0/0	0/0	3/0	4/0
*Leptoiulus proximus* (Němec, 1896)	58/1	38/2	32/4	15/0	63/1	50/6
*Megaphyllum projectum* Verhoeff, 1894	22/3	6/3	0/0	6/1	13/3	9/2
*Megaphyllum unilineatum* (C. L. Koch, 1838)	8/4	33/9	37/14	10/4	33/10	35/13
*Ommatoiulus sabulosus* (Linnaeus, 1758)	4/6	4/7	0/8	2/1	6/4	0/16
*Unciger transsilvanicus* (Verhoeff, 1899)	13/2	19/13	14/6	11/4	20/5	15/12
Opiliones						
*Dicranolasma scabrum* (Herbst, 1799)	1/0	1/0	0/0	0/0	0/0	2/0
*Nemastoma lugubre* (Müller, 1776)	1/8	2/54	0/19	1/20	1/36	1/25
*Trogulus nepaeformis* (Scopoli, 1763)	3/0	2/1	0/1	0/0	4/2	1/0
*Trogulus tricarinatus* (Linnaeus, 1767)	2/2	10/2	3/3	2/1	7/1	6/5
*Lacinius ephippiatus* (C. L. Koch, 1835)	0/10	0/11	0/53	0/9	0/24	0/41
*Lophopilio palpinalis* (Herbst, 1799)	0/13	8/19	1/25	6/7	3/16	0/34
*Oligolophus tridens* (C. L. Koch, 1836)	0/10	0/39	0/25	0/7	0/18	0/49
*Platybunus bucephalus* (C. L. Koch, 1835)	1/0	0/0	0/0	0/0	1/0	0/0
*Rilaena triangularis* (Herbst, 1799)	0/0	1/0	0/0	0/0	0/0	1/0
*Zachaeus crista* (Brullé, 1832)	16/0	28/0	47/0	5/0	22/0	64/0
*Astrobunus laevipes* (Canestrini, 1872)	3/288	7/550	5/213	3/264	9/496	3/291

## References

[ece37820-bib-0001] Abensperg‐Traun, M. , & Steven, D. (1995). The effects of pitfall trap diameter on ant species richness (Hymenoptera: Formicidae) and ant species composition of the catch in a semi‐arid eucalypt woodland. Australian Journal of Ecology, 20, 282–287. 10.1111/j.1442-9993.1995.tb00540.x

[ece37820-bib-0002] Adis, J. (1979). Problems of interpreting arthropod sampling with pitfall traps. Zoologischer Anzeiger, 202, 177–184.

[ece37820-bib-0003] Anderson, M. J. (2001). A new method for non‐parametric multivariate analysis of variance. Austral Ecology, 26, 32–46. 10.1111/j.1442-9993.2001.01070.pp.x

[ece37820-bib-0004] Anderson, M. J. (2004). DISTLM v.5: A FORTRAN computer program to calculate a distance‐based multivariate analysis for a linear model. University of Auckland.

[ece37820-bib-0005] Banerjee, B. (1970). A mathematical model on sampling diplopods using pitfall traps. Oecologia, 4, 102–105. 10.1007/BF00390617 28309038

[ece37820-bib-0006] Barber, H. S. (1931). Traps for cave‐inhabiting insects. Journal of the Elisha Mitchell Scientific Society, 46, 259–266.

[ece37820-bib-0007] Blower, J. G. (1970). The millipedes of a Cheshire wood. Journal of Zoology, 160, 455–496. 10.1111/j.1469-7998.1970.tb03092.x

[ece37820-bib-0008] Branquart, É. , Kime, R. D. , Dufrêne, M. , & Tavernier, J. (1995). Macroarthropod‐habitat relationships in oak forest in South Belgium. 1. Environments and communities. Pedobiologia, 39, 243–263. Retrieved from http://hdl.handle.net/2078.1/47993

[ece37820-bib-0009] Brennan, K. E. C. , Majer, J. D. , & Reygaert, N. (1999). Determination of an optimal pitfall trap size for sampling spiders in a Western Australian Jarrah Forest. Journal of Insect Conservation, 3, 1–11. 10.1023/A:1009682527012

[ece37820-bib-0010] Brown, G. R. , & Matthews, I. M. (2016). A review of extensive variation in the design of pitfall traps and a proposal for a standard pitfall trap design for monitoring ground‐active arthropod biodiversity. Ecology and Evolution, 6, 3953–3964. 10.1002/ece3.2176 27247760 PMC4867678

[ece37820-bib-0011] Churchill, T. B. (1993). Effects of sampling method on composition of a Tasmanian coastal heathland spider assemblage. Memoirs of Queensland Museum, 33, 475–481.

[ece37820-bib-0012] Collett, R. A. , & Fisher, D. O. (2017). Time‐lapse camera trapping as an alternative to pitfall trapping for estimating activity of leaf litter arthropods. Ecology and Evolution, 7, 7527–7533. 10.1002/ece3.3275 28944036 PMC5606863

[ece37820-bib-0013] Curtis, D. J. (1980). Pitfalls in spider community studies (Arachnida, Araneae). The Journal of Arachnology, 8, 271–280.

[ece37820-bib-0014] Dahl, F. (1896). Vergleichende untersuchungen über die lebens‐weise wirbelloser aasfresser. Sitzungberichte – Königlich Preussichen Akademie Der Wissenschaften Zu Berlin, 1, 17–30.

[ece37820-bib-0015] de Oliveira, M. P. A. , Bastos‐Pereira, R. , Torres, S. H. S. , Pereira, T. S. , Batista, F. M. , Alves, J. P. , Iniesta, L. F. M. , Bouzan, R. S. , Chagas, A. , Prous, X. , Pietrobon, T. , & Ferreira, R. L. (2019). Choosing sampling methods for Chilopoda, Diplopoda and Isopoda (Oniscidea): A case study for ferruginous landscapes in Brazilian Amazonia. Applied Soil Ecology, 143, 181–191. 10.1016/j.apsoil.2019.07.012

[ece37820-bib-0016] Dornelas, M. , Gotelli, N. J. , McGill, B. , Shimadzu, H. , Moyes, F. , Sievers, C. , & Magurran, A. E. (2014). Assemblage time series reveal biodiversity change but not systematic loss. Science, 344, 296–299. 10.1126/science.1248484 24744374

[ece37820-bib-0017] Dufrene, M. , & Legendre, P. (1997). Species assemblages and indicator species: The need for a flexible asymmetrical approach. Ecological Monographs, 67, 345–366. 10.1890/0012‐9615(1997)067[0345:SAAIST]2.0.CO;2

[ece37820-bib-0018] Fischer, M. , Bossdorf, O. , Gockel, S. , Hänsel, F. , Hemp, A. , Hessenmöller, D. , Korte, G. , Nieschulze, J. , Pfeiffer, S. , Prati, D. , Renner, S. , Schöning, I. , Schumacher, U. , Wells, K. , Buscot, F. , Kalko, E. K. V. , Linsenmair, K. E. , Schulze, E.‐D. , & Weisser, W. W. (2010). Implementing large‐scale and long‐term functional biodiversity research: The Biodiversity Exploratories. Basic and Applied Ecology, 11, 473–485. 10.1016/j.baae.2010.07.009

[ece37820-bib-0019] Franke, U. , Friebe, B. , & Beck, L. (1988). Methodisches zur ermittlung der siedlungsdichte von bodentieren aus quadratproben und barberfallen. Pedobiologia, 32, 253–264.

[ece37820-bib-0020] Gerlach, A. , Voigtländer, K. , & Heidger, C. H. M. (2009). Influences of the behaviour of epigeic arthropods (Diplopoda, Chilopoda, Carabidae) on the efficiency of pitfall trapping. Soil Organisms, 81, 773–790.

[ece37820-bib-0021] Gotelli, N. J. , & Colwell, R. K. (2001). Quantifying biodiversity: Procedures and pitfalls in the measurement and comparison of species richness. Ecology Letters, 4, 379–391. 10.1046/j.1461-0248.2001.00230.x

[ece37820-bib-0022] Gotelli, N. J. , & Colwell, R. K. (2011). Estimating species richness. In A. E. Magurran , & B. J. McGill (Eds.), Biological diversity: Frontiers in measurement and assessment (pp. 39–54). Oxford University Press.

[ece37820-bib-0023] Greenslade, P. J. M. (1964). Pitfall trapping as a method for studying populations of Carabidae (Coleoptera). Journal of Animal Ecology, 39, 301–310. 10.2307/2632

[ece37820-bib-0024] Holopainen, J. K. (1992). Catch and sex ratio of Carabidae (Coleoptera) in pitfall traps filled with ethylene glycol or water. Pedobiologia, 36, 257–261.

[ece37820-bib-0025] Hothorn, T. , Bretz, F. , & Westfall, P. (2008). Simultaneous inference in general parametric models. Biometrical Journal, 50, 34–363. 10.1002/bimj.200810425 18481363

[ece37820-bib-0026] Ilić, B. S. , Vujić, V. D. , Jovanović, Z. S. , Pavković‐Lučić, S. B. , Dudić, B. D. , Lučić, L. R. , & Makarov, S. E. (2019). Sexual dimorphism in some morphological traits of three European millipedes (Diplopoda, *Julida*, Julidae). Animal Biology, 69, 483–496. 10.1163/15707563-20191113

[ece37820-bib-0027] Inyang, U. E. , & Emosairue, S. O. (2003). Evaluation of sampling techniques for millipedes. Moor Journal of Agricultural Research, 4(2), 230–235.

[ece37820-bib-0028] Knapp, M. , Baranovská, E. , & Jakubec, P. (2016). Effects of bait presence and type of preservative fluid on ground and carrion beetle samples collected by pitfall trapping. Environmental Entomology, 45, 1022–1028. 10.1093/ee/nvw047 27260789

[ece37820-bib-0029] Knapp, M. , Knappová, J. , Jakubec, P. , Vonička, P. , & Moravec, P. (2020). Incomplete species lists produced by pitfall trapping: How many carabid species and which functional traits are missing? Biological Conservation, 245, 10.1016/j.biocon.2020.108545

[ece37820-bib-0030] Knapp, M. , & Růžička, J. (2012). The effect of pitfall trap construction and preservative on catch size, species richness and species composition of ground beetles (Coleoptera: Carabidae). European Journal of Entomology, 109, 419–426. 10.14411/eje.2012.054

[ece37820-bib-0031] Kocourek, P. , Tajovský, K. , & Dolejš, P. (2017). Millipedes of the Czech Republic: Manual for determination of the Czech millipedes (p. 256). Czech Union for Nature Conservation.

[ece37820-bib-0032] Koivula, M. , Kotze, D. J. , Hiisivuori, L. , & Rita, H. (2003). Pitfall trap efficiency: Do trap size, collecting fluid and vegetation structure matter? Entomologica Fennica, 14, 1–14. 10.33338/ef.84167

[ece37820-bib-0033] Kruskal, J. B. (1964). Multidimensional scaling by optimizing goodness of fit to a nonmetric hypothesis. Psychometrika, 29, 1–27. 10.1007/BF02289565

[ece37820-bib-0034] Lang, A. (2000). The pitfalls of pitfalls: A comparison of pitfall trap catches and absolute density estimates of epigeal invertebrate predators in arable land. Journal of Pest Science, 73, 99–106. 10.1007/BF02956438

[ece37820-bib-0035] Lindtner, J. , Gajdoš, P. , Stašiov, S. , Čiliak, M. , Pech, P. , & Kubovčik, V. (2020). Spider (Araneae) and harvestman (Opiliones) communities are structured by the ecosystem engineering of burrowing mammals. Insect Conservation and Diversity, 13, 262–270. 10.1111/icad.12382

[ece37820-bib-0036] Lövei, G. L. , & Sunderland, K. D. (1996). Ecology and behavior of ground beetles (Coleoptera: Carabidae). Annual Review of Entomology, 41, 231–256. 10.1146/annurev.en.41.010196.001311 15012329

[ece37820-bib-0037] Luff, M. L. (1975). Some features influencing the efficiency of pitfall traps. Oecologia, 4, 345–357. 10.1007/bf00348110 28309246

[ece37820-bib-0038] Magurran, A. E. , Baillie, S. R. , Buckland, S. T. , Dick, J. M. , Elston, D. A. , Scott, E. M. , Smith, R. I. , Somerfield, P. J. , & Watt, A. D. (2010). Long‐term datasets in biodiversity research and monitoring: Assessing change in ecological communities through time. Trends in Ecology and Evolution, 25, 574–582. 10.1016/j.tree.2010.06.016 20656371

[ece37820-bib-0039] Majer, J. D. (1997). The use of pitfall traps for sampling ants – A critique. Memoirs of Museum Victoria, 56, 323–329. 10.24199/j.mmv.1997.56.20

[ece37820-bib-0040] Martens, J. (1978). Weberknechte, Opiliones – Spinnentiere, Arachnida. In K. Senglaub , H. J. Hannemann , & H. Shumann (Eds.), Die Tierwelt Deutschlands (Vol. 64). VEB G. Fischer Verlag.

[ece37820-bib-0041] McArdle, B. H. , & Anderson, M. J. (2001). Fitting multivariate models to community data: A comment on distance‐based redundancy analysis. Ecology, 82, 290–297. 10.1890/0012‐9658(2001)082[0290:fmmtcd]2.0.CO;2

[ece37820-bib-0042] McCravy, K. W. , & Willand, J. E. (2007). Effects of pitfall trap preservative on collections of carabid beetles (Coleoptera: Carabidae). The Great Lakes Entomologist, 40, 154–165. Retrieved from https://scholar.valpo.edu/tgle/vol40/iss2/6

[ece37820-bib-0043] Miklós, L. (Ed.) (2003). Landscape Atlas of the Slovak Republic (1st ed.). Ministry of Environment of the Slovak Republic, Slovak Environmental Agency.

[ece37820-bib-0044] Müller, J. K. (1984). Die bedeutung der fallenfang – methode für die lösung ökologischer fragestellungen. Zoologische Jahrbücher. Abteilung Für Systematik, Geographie und Biologie der Tiere, 111, 281–305.

[ece37820-bib-0045] Pekár, S. (2002). Differential effects of formaldehyde concentration and detergent on catching efficiency of surface active arthropods by pitfall traps. Pedobiologia, 46, 539–547. 10.1078/0031-4056-00158

[ece37820-bib-0046] Petruška, F. (1969). On possibility of escape of the various components of the epigeic fauna of the fields from pitfall traps containing Formalin. Acta Universitatis Palackianae Olomucensis, Facultas Rerum Naturalium, 31, 99–124.

[ece37820-bib-0047] Pinto‐da‐Rocha, R. , Machado, G. , & Gibert, G. (2007). Harvestmen: The biology of opiliones (p. 597). Harvard University Press.

[ece37820-bib-0048] Prasifka, J. R. , Lopez, M. D. , Hellmich, R. L. , Lewis, L. C. , & Dively, G. P. (2007). Comparison of pitfall traps and litter bags for sampling ground‐dwelling arthropods. Journal of Applied Entomology, 131, 115–120. 10.1111/j.1439-0418.2006.01141.x

[ece37820-bib-0049] Privet, K. , Vedel, V. , Fortunel, C. , Orivel, J. , Martinez, Q. , Cerdan, A. , Baraloto, C. H. , & Pétillon, J. (2020). Relative efficiency of pitfall trapping vs. nocturnal hand collecting in assessing soil‐dwelling spider diversity along a structural gradient of neotropical habitats. Diversity, 12, 81. 10.3390/d12020081

[ece37820-bib-0050] R Core Team (2018). R: A language and environment for statistical computing. R Foundation for Statistical Computing. Retrieved from https://www.R‐project.org/

[ece37820-bib-0051] Roberts, D. W. (2016). Labdsv: Ordination and multivariate analysis for ecology. R package version 1.8‐0. Retrieved from https://CRAN.R‐project.org/package=labdsv

[ece37820-bib-0052] Saska, P. , van der Werf, W. , Hemerik, L. , Luff, M. L. , Hatten, T. D. , & Honek, A. (2013). Temperature effects on pitfall catches of epigeal arthropods: A model and method for bias correction. Journal of Applied Ecology, 50, 181–189. 10.1111/1365-2664.12023 PMC3607414 23539634

[ece37820-bib-0053] Schmidt, M. H. , Clough, Y. , Schulz, W. , Westphalen, A. , & Tscharntke, T. (2006). Capture efficiency and preservation attributes of different fluids in pitfall traps. The Journal of Arachnology, 34, 159–162. 10.1636/T04-95.1

[ece37820-bib-0054] Sechterová‐Špičáková, E. (1989). Spiders (Araneida) and harvestmen (Opiliones) in the groves II. Acta Universitatis Palackianae Olomoucensis, Facultas Rerum Naturalium, Biologica, 29, 165–184.

[ece37820-bib-0055] Siewers, J. , Schirmel, J. , & Buchholz, S. (2014). The efficiency of pitfall traps as a method of sampling epigeal arthropods in litter rich forest habitats. European Journal of Entomology, 111, 69–74. 10.14411/eje.2014.008

[ece37820-bib-0056] Skvarla, M. J. , Larson, J. L. , & Dowling, A. P. G. (2014). Pitfalls and preservatives: A review. Journal of Entomological Society of Ontario, 145, 15–43.

[ece37820-bib-0057] Southwood, T. R. E. (1978). Relative Methods of Population Measurement and the Derivation of Absolute Estimates: Land Pitfall and Other Traps Ecological Methods with Particular Reference to Insect Populations (2nd ed.). Chapman and Hall.

[ece37820-bib-0058] Spence, J. R. , & Niemelä, J. (1994). Sampling carabid assemblages with pitfall traps: The madness and the method. Canadian Entomologist, 126, 881–894. 10.4039/Ent126881-3

[ece37820-bib-0059] Stammer, H. (1948). Die bedeutung der aethylen‐glykolfallen für tierökologiesche und phänologische untersuchungen. Verhandlungen Der Deutschen Zoologischen Gesellschaft, 387–391.

[ece37820-bib-0060] Stanová, V. , & M. Valachovič (Eds.) (2002). Catalogue of biotopes in Slovakia. Daphne – Institute of Applied Ecology.

[ece37820-bib-0061] Stašiov, S. (2015). Ecology of soil organisms (Soil Animals). University Textbook. Technical University in Zvolen.

[ece37820-bib-0062] Štrobl, M. , Saska, P. , Seidl, M. , Kocian, M. , Tajovský, K. , Řezáč, M. , Skuhrovec, J. , Marhoul, P. , Zbuzek, B. , Jakubec, P. , Knapp, M. , & Kadlec, T. (2019). Impact of an invasive tree on arthropod assemblages in woodlots isolated within an intensive agricultural landscape. Diversity and Distribution, 25, 1800–1813. 10.1111/ddi.12981

[ece37820-bib-0063] Thiele, H. U. (1977). Carabid Beetles in Their Environment: A study on habitat selection by adaptations in physiology and behavior. Springer.

[ece37820-bib-0064] Topping, C. J. , & Sunderland, K. D. (1992). Limitations to the use of pitfall traps in ecological studies exemplified by a study of spiders in a field of winter wheat. Journal of Applied Ecology, 29, 485–491. 10.2307/2404516

[ece37820-bib-0065] Törmälä, T. (1982). Evaluation of five methods of sampling field layer arthropods, particularly the leafhopper community, in grassland. Annales Entomologicae Fennicae, 48, 1–16.

[ece37820-bib-0066] Tourinho, A. L. , & Lo‐Man‐Hung, N. (2021). Standardized sampling methods and protocols for harvestman and spider assemblages. In J. C. Santos , & G. W. Fernandes (Eds.), Measuring arthropod biodiversity (pp. 365–400). Springer. 10.1007/978-3-030-53226-0_15

[ece37820-bib-0067] Ward, D. F. , New, T. R. , & Yen, A. L. (2001). Effects of pitfall trap spacing on the abundance, richness and composition of invertebrate catches. Journal of Insect Conservation, 5, 47–53. 10.1023/A:1011317423622

[ece37820-bib-0068] Wijnhoven, H. (2009). De Nederlandse hooiwagens (Opiliones). Entomologische Tabellen, 3, 1–118.

[ece37820-bib-0069] Work, T. T. , Buddle, C. M. , Korinus, L. M. , & Spence, J. R. (2002). Pitfall trap size and capture of three taxa of litter‐dwelling arthropods: Implications for biodiversity studies. Environmental Entomology, 31, 438–448. 10.1603/0046-225X-31.3.438

[ece37820-bib-0070] Zou, Y. , Feng, J. , Xue, D. , Sang, W. , & Axmacher, J. C. (2012). Comparison of terrestrial arthropod sampling methods. Journal of Resources and Ecology, 3, 174–182. 10.5814/j.issn.1674-764x.2012.02.010

